# An artificial neural-network approach to identify motor hotspot for upper-limb based on electroencephalography: a proof-of-concept study

**DOI:** 10.1186/s12984-021-00972-7

**Published:** 2021-12-20

**Authors:** Ga-Young Choi, Chang-Hee Han, Hyung-Tak Lee, Nam-Jong Paik, Won-Seok Kim, Han-Jeong Hwang

**Affiliations:** 1 Department of Electronics and Information Engineering, Korea University, Sejong, 30019 Republic of Korea; 2 Department of Software, College of Software Convergence, Dongseo University, Busan, 47011 South Korea; 3 Interdisciplinary Graduate Program for Artificial Intelligence Smart Convergence Technology, Korea University, Sejong, 30019 South Korea; 4 Department of Rehabilitation Medicine, Seoul National University College of Medicine, Seoul National University Bundang Hospital, Seongnam-si, 13620 Republic of Korea

**Keywords:** Motor hotspot, Electroencephalography, Transcranial electrical stimulation, Machine learning, Artificial neural-network

## Abstract

**Background:**

To apply transcranial electrical stimulation (tES) to the motor cortex, motor hotspots are generally identified using motor evoked potentials by transcranial magnetic stimulation (TMS). The objective of this study is to validate the feasibility of a novel electroencephalography (EEG)-based motor-hotspot-identification approach using a machine learning technique as a potential alternative to TMS.

**Methods:**

EEG data were measured using 63 channels from thirty subjects as they performed a simple finger tapping task. Power spectral densities of the EEG data were extracted from six frequency bands (delta, theta, alpha, beta, gamma, and full) and were independently used to train and test an artificial neural network for motor hotspot identification. The 3D coordinate information of individual motor hotspots identified by TMS were quantitatively compared with those estimated by our EEG-based motor-hotspot-identification approach to assess its feasibility.

**Results:**

The minimum mean error distance between the motor hotspot locations identified by TMS and our proposed motor-hotspot-identification approach was 0.22 ± 0.03 cm, demonstrating the proof-of-concept of our proposed EEG-based approach. A mean error distance of 1.32 ± 0.15 cm was measured when using only nine channels attached to the middle of the motor cortex, showing the possibility of practically using the proposed motor-hotspot-identification approach based on a relatively small number of EEG channels.

**Conclusion:**

We demonstrated the feasibility of our novel EEG-based motor-hotspot-identification method. It is expected that our approach can be used as an alternative to TMS for motor hotspot identification. In particular, its usability would significantly increase when using a recently developed portable tES device integrated with an EEG device.

## Introduction

Motor impairment is a frequent symptom occurring after neurological disorders, such as stroke and Parkinson’s disease [1–3]. Although motor functions are not completely restored after neurological disorders, continuous rehabilitation is necessary to prevent muscle loss and the retrogression of intact motor functions. Conventional motor rehabilitation interventions involve specific movements related to the affected limbs, which are enforced by a therapist or an assistive rehabilitation device.

Recently, transcranial electrical stimulation (tES) capable of modulating cortical excitability using a weak electrical current has been introduced for motor rehabilitation, and its positive effects have been proven in many interventional studies even though the mechanisms have not yet been fully understood [4–10]. For example, one study showed that anodal transcranial direct current stimulation (tDCS) on the ipsilesional primary motor cortex could improve overall motor functions of the upper limbs in stroke patients, and its effects persisted for at least 3 months post-intervention [11]. Another study also demonstrated the positive effects of transcranial alternating current stimulation (tACS) on motor performance improvements in Parkinson’s disease [12].

To maximize the positive effects of tES on motor rehabilitation, it is important to find an optimal tES target location on the scalp. The lesional primary motor area (M1) and its contralateral motor area have been traditionally used as stimulation target locations, to which anodal and cathodal tES electrodes are applied, respectively [13–15]. A cathodal tES electrode is sometimes applied to the contralateral supraorbital area of the lesional hemisphere instead of the contralateral motor area, called the M1-supraorbital prominence [15–17]. Identifying M1 can be performed by either the international 10–20 coordinate system used for electroencephalography (EEG) measurements or motor evoked potentials (MEPs), induced by transcranial magnetic stimulation (TMS). The former method uses C3 on the left hemisphere or C4 on the right hemisphere as the M1 location, depending on the lesional side [13, 18]. However, as the international 10–20 coordinate system does not consider inter-subject variability in cortical anatomy, the M1 location (C3 or C4) found by the international 10–20 coordinate system might not be an appropriate tES target location for motor rehabilitation. In particular, patients with neurological disorders, who are the main recipients of tES treatment, have shown significant changes in cortical morphologies owing to neural reorganization and plasticity after the occurrence of neurological disorders, such as stroke and cerebral palsy [19], requiring better methods to find individualized tES target locations more precisely.

As an alternative to the international 10–20 coordinate system, many studies have used TMS-induced MEP to find an individualized tES target location, called motor hotspot, for motor rehabilitation. This has been done because applying tES to the motor hotspot found by TMS-induced MEP could provide focal and accurate neuromodulatory effects on the motor network [20–22]. TMS is a useful tool to find motor hotspots, but requires a relatively bulky device and a somewhat cumbersome procedure accompanying the empirical judgment of a technician. Moreover, another device that measures MEP is required to find an individual motor hotspot using TMS.

In this study, we propose a novel alternative to TMS for motor hotspot identification based on EEG data measured during a simple finger-tapping motor task. A machine learning technique based on a multilayer perceptron artificial neural network (ANN) was applied to EEG power spectral density (PSD) features to localize individualized motor hotspots. The motor hotspot positions estimated by our proposed EEG-based machine-learning approach were then compared to those found by the traditional TMS-induced MEP to verify the feasibility of our approach. Preliminary results have been shown in [23], where we simply investigated the possibility of our proposed EEG-based motor-hotspot-identification approach with a small number of subjects (10 subjects) by employing only right hand. In this study, we significantly extended the preliminary work by investigating not only the feasibility of our proposed EEG-based motor-hotspot-identification approach using more EEG data (30 subjects), but also its practical feasibility with a reduced number of EEG electrodes and the EEG data for both hands.

## Methods

### Subjects

Thirty healthy subjects (10 females and 20 males; 25 ± 1.39 years; all right-handed) participated in this study, and they had no history of psychiatric diseases that might affect research results. All subjects received the information about the details of the experimental procedure and signed an informed consent for participation in the study. Appropriate monetary compensation (about $ 10 per hour) for their participation was provided after the experiment. This study was approved by the Institutional Review Board (IRB) of Kumoh National Institute of Technology (No. 6250) and was conducted in accordance with the principles of the declaration of Helsinki.

### Motor hotspot identification by TMS-induced MEP

Before measuring the EEG data, the motor hotspots of both hands were identified for each subject using the MEPs of the first dorsal interosseous (FDI) muscle. To this end, Ag–AgCl disposable electrodes were attached to the FDI muscles of both hands to measure the MEPs (actiCHamp, Brain Products GmbH, Gilching, Germany). We applied a series of single-pulse TMS to the contralateral motor cortex by slightly moving the TMS position during the resting state (REMED., Daejeon, Korea), and first determined motor hotspot candidates showing a MEP amplitude over 50 μV accompanying the movement of a target muscle (FDI). A motor hotspot candidate showing the largest MEP amplitude was then tested whether this candidate shows an amplitude of over 50 μV at least more than 5 of 10 single-pulse TMS. If the motor hotspot candidate met the criterion of motor hotspot identification, this motor hotspot candidate was finally determined as the motor hotspot of a subject. Otherwise, we tested the next motor hotspot candidate that showed the second largest MEP to check whether this candidate met the criterion of motor hotspot identification, which was iterated until a motor hotspot candidate met the mentioned criterion [24–26]. The motor hotspot locations were identified once for each subject, and they were marked in a 3D coordinate system based on the vertex (Cz in the international 10–20 system) using a 3D digitizer (Polhemus Inc., Colchester, Vermont, USA). The motor hotspot locations transformed into the 3D coordinate system were used as the ground truth to compare with those estimated by our EEG-based motor-hotspot-identification approach.

### EEG recording

**Fig. 1:**
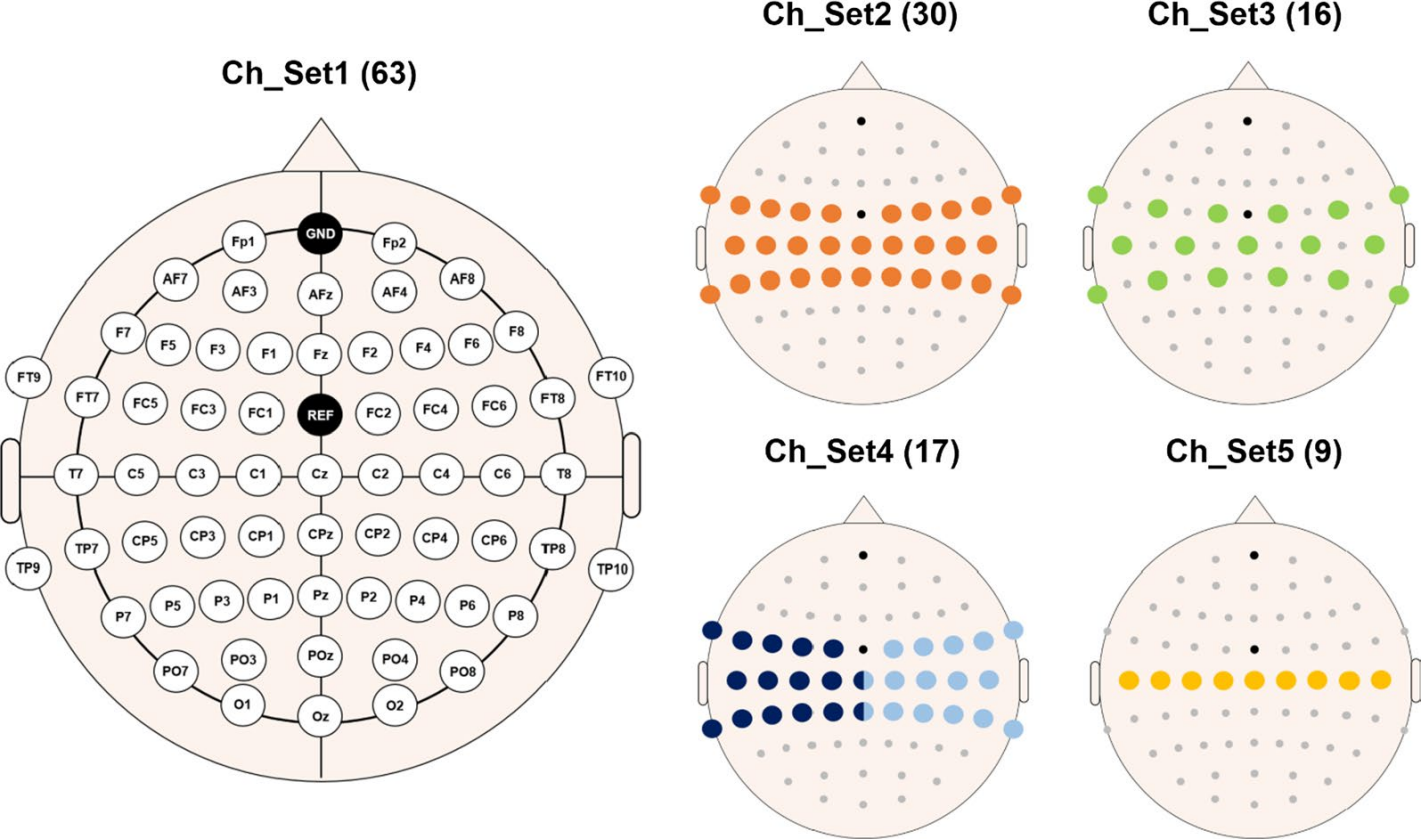
Electrode sites used for EEG data measurement. Five different channel sets were used for data analysis in order to study the impact of the number of channels on the error distance of motor hotspot location. Channels denoted by different colors in Ch_Set4 represent those selected on the contralateral motor cortex for the data analysis of each hand. The numbers in parentheses indicate the numbers of channels for the corresponding channel set

**Fig. 2:**
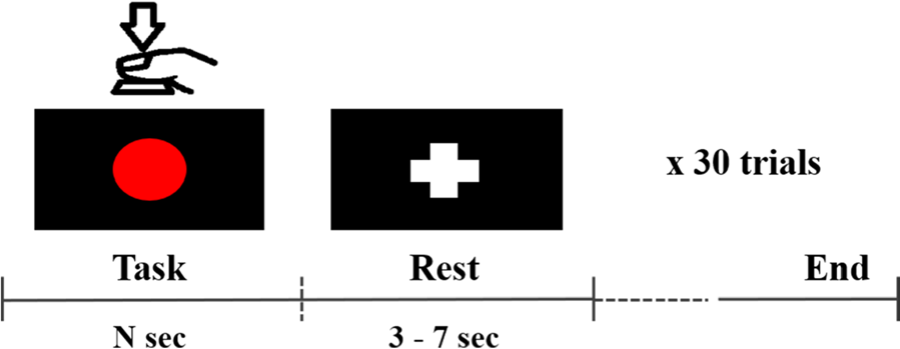
Experimental paradigm used in this study. Each subject presses a space bar whenever the red circle is presented in the middle of a screen. The red circle is maintained until the subject presses the space bar. “N sec” means different response times between trials. After the task period, the fixation (‘+’) mark is presented to indicate a rest period. The average response time across all subjects was about 690 ± 25 ms for both hands (680 ± 30 ms for right hand and 710 ± 20 ms for left hand)

After identifying the individual motor hotspots using TMS for both hands, task-related EEG data were measured using 63 EEG electrodes attached to the scalp based on the international 10–20 system (Fig. 1). The ground and reference electrodes were attached to Fpz and FCz, respectively. The positions of the EEG electrodes were marked in the 3D coordinate system as for the motor hotspot locations identified by TMS-induced MEPs, and they were used to transform the 3D coordinate information of motor hotspot locations found by TMS into our 3D coordinate system with the origin (0, 0, 0) at Cz in the international 10–20 system. The EEG data were sampled at 1000 Hz using a multi-channel EEG acquisition system (actiCHamp, Brain Products GmbH, Gilching, Germany) while the subjects were performing a simple finger-tapping motor task. Figure 2 shows the experimental paradigm of this study. Each subject performed to press a button 30 times using their index fingers whenever a red circle was presented in the center of a monitor; two EEG measurement sessions were independently conducted using the left and right index fingers, respectively, resulting in a total of 60 trials for both hands (30 trials for each hand). The subjects were given sufficient rest whenever they wanted during the experiment to avoid fatigue. Moreover, they were instructed to remain relaxed during the experiment without any movement to prevent unwanted physiological artifacts. Because we carried out preliminary experiments with the first two subjects to validate our experimental paradigm before the main experiment, the right hand was only employed for these two subjects. In addition, we excluded the EEG data of one subject for both hands, and those of the left hand for other three subjects due to high contamination of EEG data caused by physiological artifacts. Thus, 29 and 25 EEG datasets were used for the right and left hands, respectively, for data analysis.

### EEG-based motor hotspot identification

EEG signal preprocessing was performed using the EEGLAB toolbox based on MATLAB 2017b (MathWorks, Natick, MA, USA). The raw EEG data were first down-sampled into 200 Hz to which we sequentially applied common average reference and bandpass filtering between 0.5 and 50.5 Hz (zero-phase 3rd-order Butterworth filter). Independent component analysis (ICA) was then applied to the filtered EEG data to remove physiological artifacts, where an average of 20.0 ± 6.66 components was removed across the subjects (median = 21). As mentioned above, from visual inspection we excluded the EEG data significantly contaminated after ICA application (EEG data of one subject for both hands and those of the left hand for other three subjects were excluded). No other datasets or trials/channels of the remaining datasets were excluded, for which we cross-checked EEG patterns before, during, and after ICA application based on the EEGLAB guideline with a conservative rejection criterion [27].

**Fig. 3:**
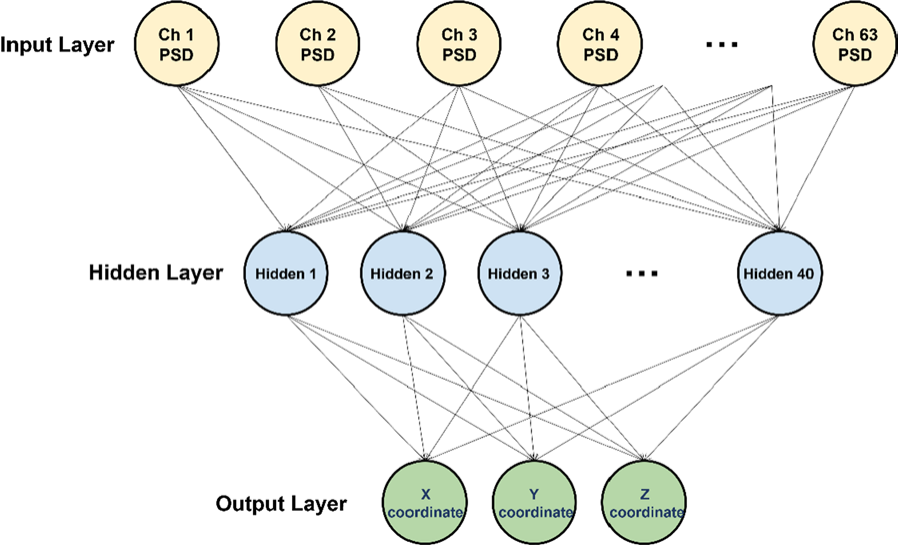
Architecture of an artificial neural network (ANN) model used for EEG-based motor hotspot identification. The numbers of input nodes, hidden nodes, and output nodes were 63 (PSD features of 63 channels), 40 (empirically selected), and 3 (3D motor hotspot coordinates), respectively

After preprocessing, we epoched EEG data between − 0.5 and 0.5 s based on the key press point for each trial to extract motor-related EEG features. PSDs of each EEG epoch (trial) were estimated in six frequency bands (delta: 1–4 Hz, theta: 4–8 Hz, alpha: 8–13 Hz, beta: 13–30 Hz, gamma: 30–50 Hz, and full: 1–50 Hz) using the fast Fourier transform (FFT). A frequency resolution was 1 Hz for PSD estimation because a 1-s epoch was used for FFT. An ANN was used to estimate the motor hotspot location using PSD features where a tenfold cross-validation was performed with early stopping to prevent overfitting; all subjects’ data (30 trials for each subject) were pooled together and they were randomly split into tenfolds, where ninefolds (training data) were used to train an ANN model whereas the remaining fold (test data) was used to evaluate its performance, which was iterated until each fold was tested once. The ratio of training data and test data was equally kept at 9:1 for each subject’s data. The mentioned cross-validation scheme was introduced because we regarded our machine learning problem as a multi-class machine learning problem due to different class labels (individual motor hotspot locations) for each subject. The PSD features of each channel and the 3D location coordinates of the motor hotspot identified by TMS-induced MEP (ground truth) in training data were used to train an ANN model. The PSD features of each channel in test data were then fed into the trained ANN model, and their corresponding outputs were 3D motor hotspot coordinates estimated using the trained ANN model. The numbers of input nodes, hidden nodes, and output nodes were 63 (PSD features of 63 channels), 40 (empirically selected), and 3 (3D motor hotspot coordinates), respectively, as shown in Fig. 3. We calculated the Euclidean distance between the 3D motor hotspot coordinates identified by TMS and EEG PSD features to quantitatively estimate the error distance of our EEG-based motor-hotspot-identification approach. The data analysis was independently performed for each of the six EEG frequency bands.

To investigate the impact of the number of channels on the error distance, we repeatedly performed the mentioned analysis by reducing the number of channels, in particular focusing on the channels attached to the motor area (Fig. 1). For this investigation, only gamma-band PSD features (30–50 Hz) were used because they showed the best mean error distance when using all 63 channels. Note that gamma band is also closely related to a motor task along with alpha and beta frequency bands [28–31].

## Results

**Fig. 4:**
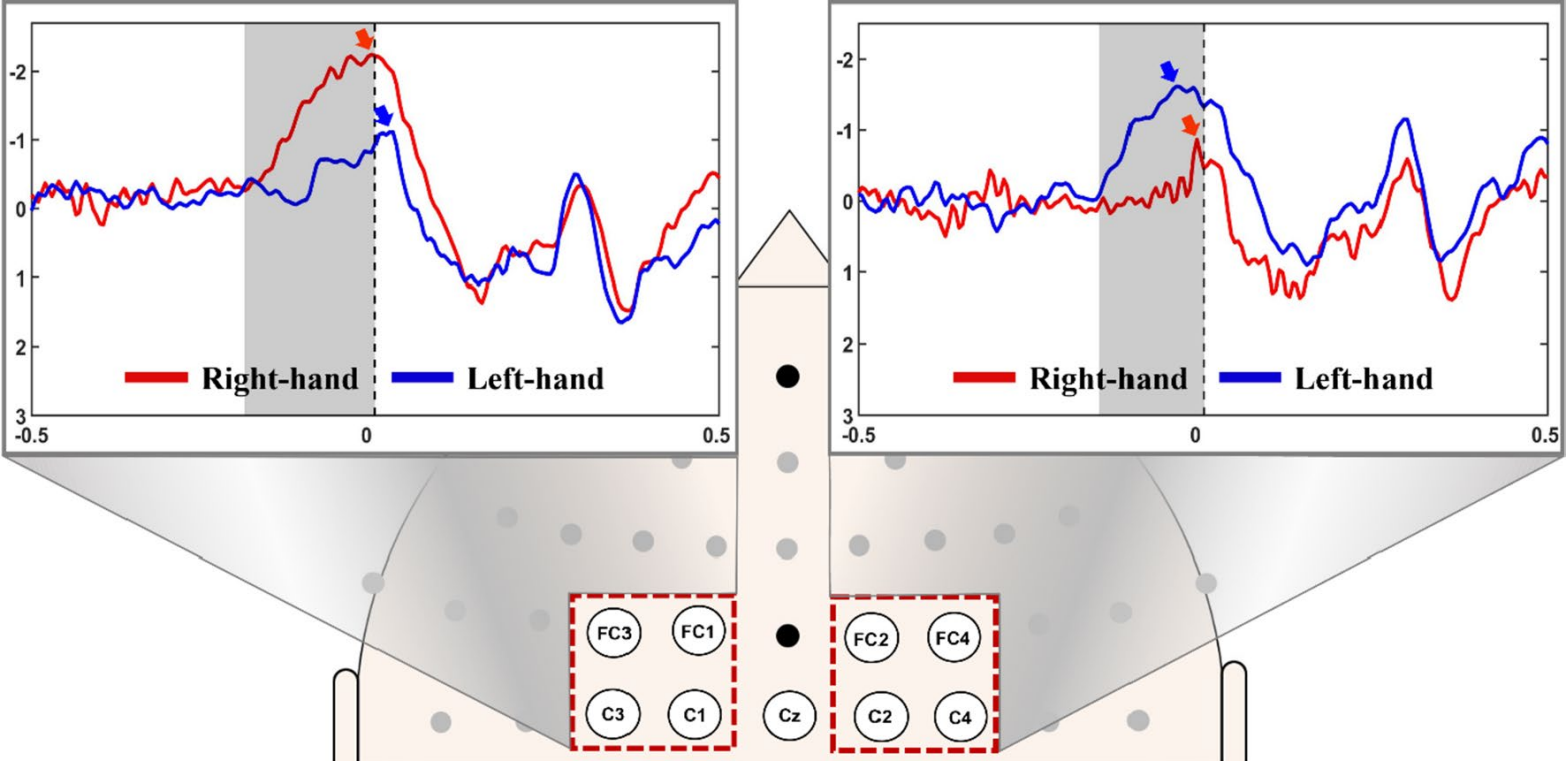
Grand averaged movement-related event-related potential (ERP) patterns of all subjects on both hemispheres. Contralateral movement-related ERPs are clearly observed. A gray area and an arrow represent the movement-related ERP period and the peak of movement-related ERPs, respectively. Note that the sign of y-axis is inverted

Figure 4 shows the grand-average event-related potential (ERP) measured during finger tapping between − 0.5 and 0.5 s based on the task onset, which was obtained by averaging the movement-related ERPs of four channels located on each hemisphere of the motor cortex to confirm the reliability of our EEG data (baseline period: − 1 to − 0.5 s). Note that the movement-related ERP obtained by averaging 20 trials are enough to investigate movement-related EEG patterns [32] (30 trials were measured in this study for each hand). Movement-related ERP was clearly observed on the motor cortex during finger tapping for both hands, and, in particular, stronger movement-related ERP was observed on the contralateral motor area [33, 34]. The peak amplitudes and latencies of movement-related ERP of contralateral and ipsilateral motor areas were − 2.24 with − 320 ms and − 1.11 with − 390 ms for left hand, and those were − 1.85 with − 260 ms and − 0.82 with − 280 ms for right hand, respectively. The latency was estimated based on the time that starts movement-related ERP (beginning of the gray area in Fig. 4) based on the onset time.

**Fig. 5:**
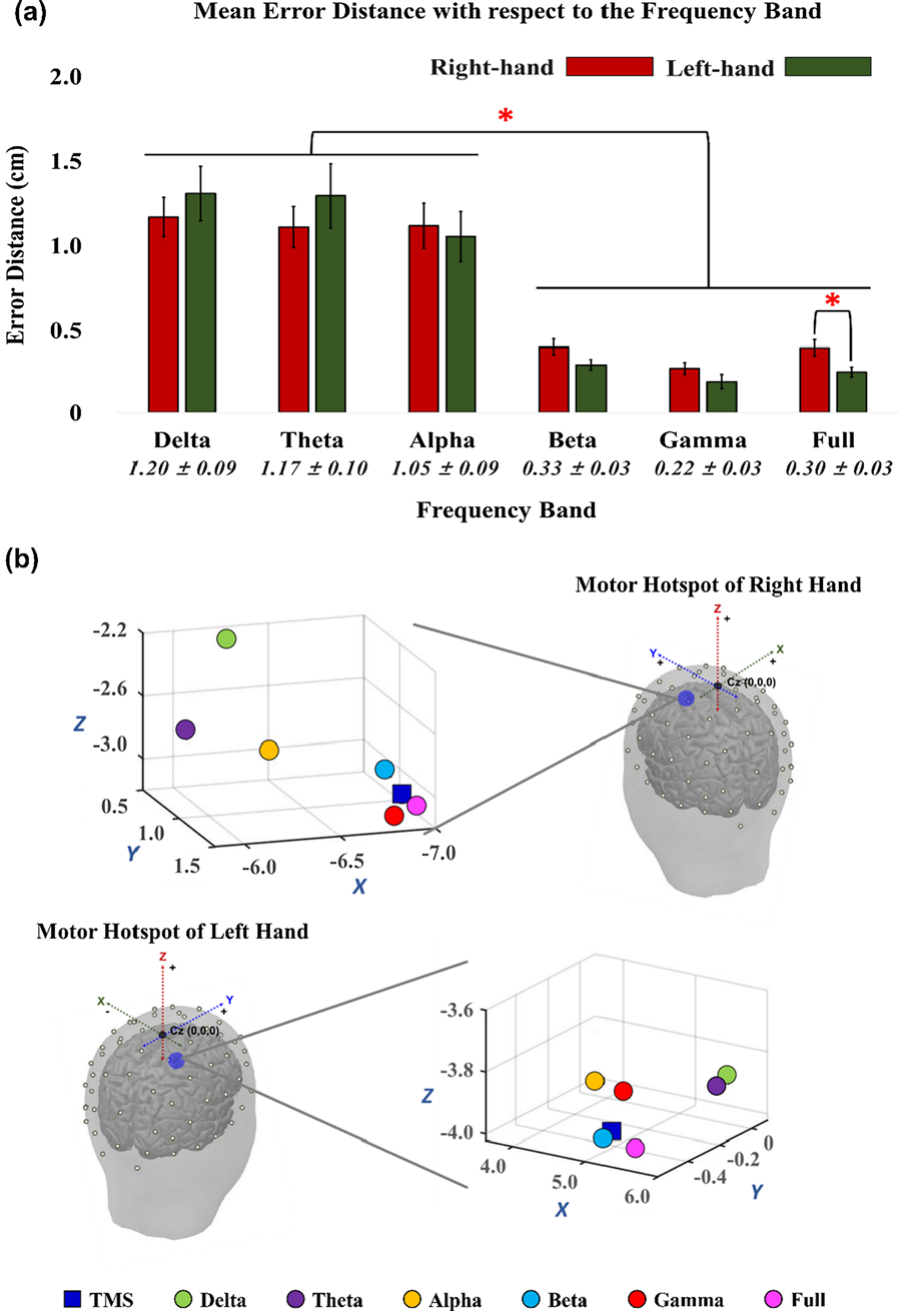
**a** Mean error distances between the motor hotspot locations identified by TMS-induced MEP and our EEG-based machine learning approach with respect to the frequency band (RM-ANOVA with Bonferroni corrected *p*-value < 0.05: delta = theta = alpha > beta = gamma = full for both hands). The numbers below the bar graphs represent the mean error distances of each frequency band and their standard errors denoted by error bars. The standard deviations of the mean error distances are ± 0.70 for delta band, ± 0.79 for theta band, ± 0.72 for alpha band, ± 0.21 for beta band, ± 0.20 for gamma band, and ± 0.22 for full band, respectively. No significant difference was observed between the left and right hands for all the frequency bands in terms of the error distance (paired t-test *p* > 0.05), except the full band (right hand > left hand). **b** A representative example showing the 3D locations of the motor hotspots identified by TMS-induced MEP (blue rectangle) and our EEG-based approach with respect to the frequency band. The X, Y, and Z coordinates correspond to the left/right, posterior/anterior, and ventral/dorsal dimension, respectively, based on the Cz (origin: 0, 0, 0)

Figure 5a shows the mean error distances of the motor hotspot locations estimated by our EEG-based machine learning approach for each hand with respect to the frequency band when using all 63 channels. The PSD features of the relatively high frequency bands (beta, gamma, and full bands) resulted in significantly lower mean error distances than those of the low frequency bands (delta, theta, and alpha) for both hands (RM-ANOVA with Bonferroni corrected *p*-value < 0.05: delta = theta = alpha > beta = gamma = full). No significant difference was observed between the left and right hand for all the frequency bands in terms of the error distance (paired t-test *p* > 0.05), except for the full band (right hand > left hand). To match the number of samples for the left and right hand in the statistical test, we used 25 subjects’ datasets that fully had both left- and right-hand EEG datasets. Figure 5b shows a representative example of a single subject displaying the 3D locations of the motor hotspot identified by TMS (blue rectangle) and those of our EEG-based approach for both hands with respect to the frequency band. The motor hotspots estimated using the relatively high frequency bands were found closer to the ground truth motor hotspot identified by TMS, as compared to those estimated using the low frequency bands.

**Fig. 6:**
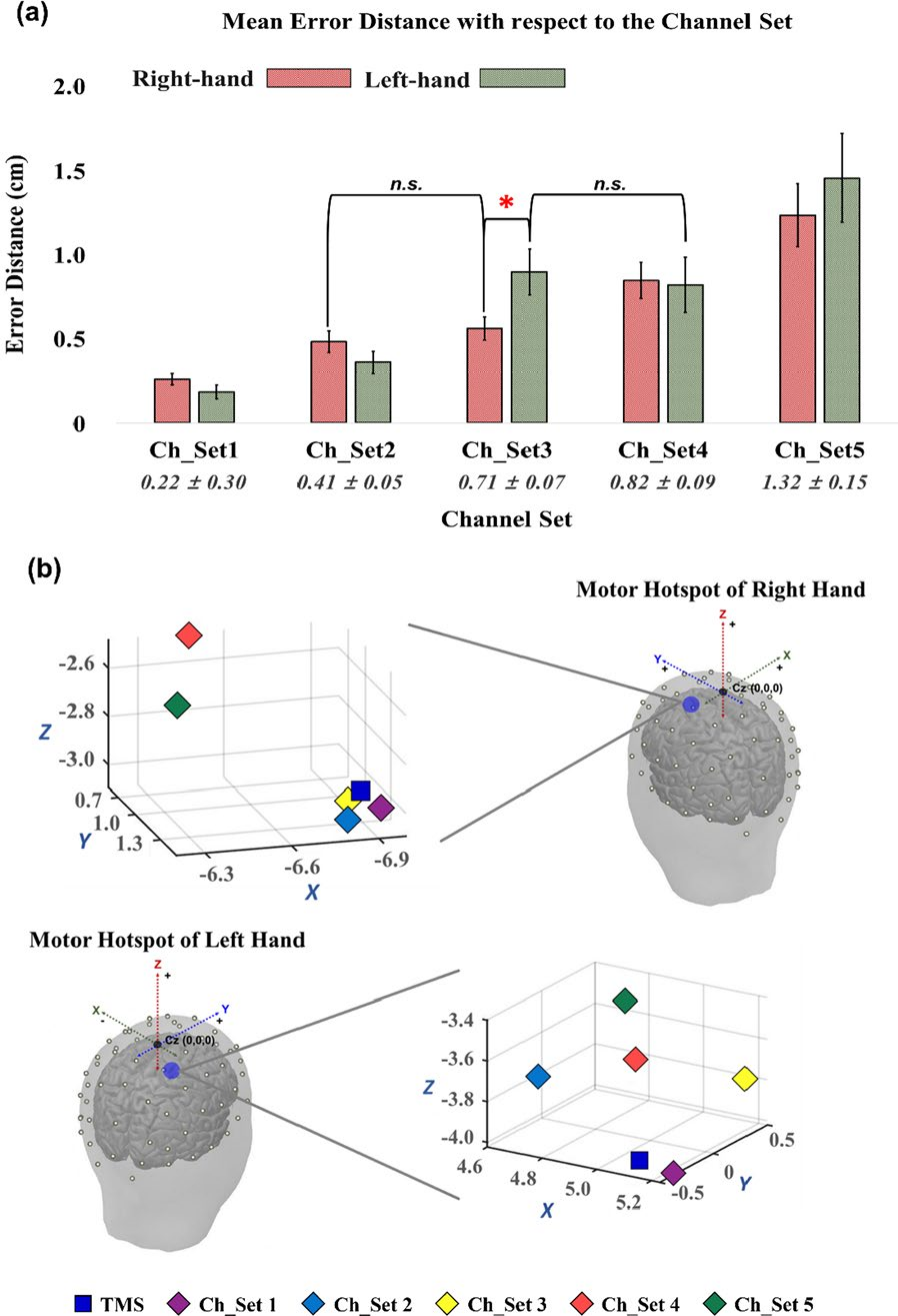
**a** Mean error distances between the motor hotspot locations identified by TMS-induced MEP and our EEG-based machine learning approach with respect to the number of channels (RM-ANOVA with Bonferroni corrected *p*-value < 0.05: Ch_Set5 > Ch_Set4 > Ch_Set3 = Ch_Set2 > Ch_Set1 for the right hand and Ch_Set5 > Ch_Set4 = Ch_Set3 > Ch_Set2 > Ch_Set1 for the left hand). The numbers below the bar graphs represent the mean error distances of each channel set and their standard errors denoted by error bars. The standard deviations of the mean error distances are ± 0.20 for Ch_Set1, ± 0.33 for Ch_Set2, ± 0.54 for Ch_Set3, ± 0.69 for Ch_Set4, ± 1.14 for Ch_Set5, respectively. No significant difference was observed between the left and right hand for all channel sets in terms of the error distance (paired t-test *p* > 0.05), except Ch_Set3 (left hand > right hand). The abbreviation, *n.s.,* means no significant difference. **b** A representative example showing the 3D locations of the motor hotspots identified by TMS (blue rectangle) and the EEG-based approach with respect to the number of channels. The X, Y, and Z coordinates correspond to the left/right, posterior/anterior, and ventral/dorsal dimension, respectively, based on the Cz (origin: 0, 0, 0)

Figure 6a presents the mean error distances of the motor hotspot locations estimated by our EEG-based approach for each hand with respect to the number of channels. In general, the mean error distance monotonically and significantly increased as the number of channels decreased (RM-ANOVA with Bonferroni corrected *p*-value < 0.05: Ch_Set5 > Ch_Set4 > Ch_Set3 = Ch_Set2 > Ch_Set1 for the right hand and Ch_Set5 > Ch_Set4 = Ch_Set3 > Ch_Set2 > Ch_Set1 for the left hand). No significant difference was observed between the left and right hand for all channel sets in terms of the error distance (paired t-test *p* > 0.05), except Ch_Set3 (left hand > right hand). Figure 6b shows the 3D locations of the motor hotspots identified by TMS and our EEG-based approach with respect to the number of channels, showing a similar trend to the result shown in Fig. 6a.

## Discussion

In this study, we proposed a novel EEG-based motor-hotspot-identification method using an ANN as a potential alternative to TMS to determine a tES target location for motor rehabilitation. A minimum mean error distance of 0.22 ± 0.03 cm was attained when using the gamma band PSD information extracted using all 63 channels, demonstrating the proof-of-concept of the proposed EEG-based motor-hotspot-identification method. An EEG device is required for the use of the proposed motor-hotspot-identification approach based on a machine learning technique. In recent years, commercial companies produced a portable tES-EEG device (e.g., Starstim by Neuroelectrics in 2012) and an tES device that can be integrated with an EEG device (e.g., M × N-5 and M × N-9 HD-tES by Soterix Medical in 2014), which could facilitate the use of our motor-hotspot-identification approach without using TMS.

TMS has been most widely used to identify motor hotspots, and TMS-based motor hotspots are used as target locations for tES application [20–22]. In order to find a motor hotspot using TMS for motor rehabilitation, patients with neurological disorders should visit a hospital equipped with a TMS and EMG devices, which is inconvenient for patients with impaired mobility who are main targets of tES for motor rehabilitation (e.g., stroke patients). Inter- and intra-subject variability of TMS-induced MEP is another problem when finding a motor hotspot [35, 36], requiring the experienced operator as well as making the time of finding a motor hotspot inconsistent, e.g., less than 10 min or more than 30 min for best or worst cases, respectively, based on our empirical investigation. Moreover, a biosignal (EMG) measurement device is additionally required for MEP measurement during TMS application. Our proposed EEG-based motor-hotspot-identification approach can be a promising alternative to the classical TMS-induced MEP approach in terms of usability and economics because our machine learning approach can automatically identify motor hotspot using EEG data measured during simple finger tapping taken less than several min without using a relatively bulky and expensive TMS device compared to EEG devices. Note that EEG can be easily set-up with tES using such the mentioned portable tES-EEG device, thereby simultaneously enabling automatic motor hotspot identification without empirical judgement and tES application together. Taken together, our proposed motor-hotspot-identification strategy can provide patients with impaired mobility with higher usability without periodic hospital visit, and it is also economically beneficial by replacing a TMS device with a relatively economical EEG device. Note that the introduction of low-cost, easy-to-use, portable EEG devices has been gradually increasing, and we showed that EEGs measured only over the motor cortex might be used to find individual motor hotspots. It is expected that the mentioned advantages of our proposed EEG-based motor-hotspot-identification approach could facilitate home-based motor rehabilitation using tES technique for patients with impaired mobility.

To check the practical feasibility of the proposed motor-hotspot-identification method, the effect of the number of channels was investigated using the gamma band PSD features. As expected, the lowest mean error was obtained using all channels, and the mean error distance increased as the number of channels decreased (Fig. 6). We obtained a mean error distance of less than 1 cm with a number of channels above 17 (Ch_Set4) broadly attached on the motor cortex. In addition, a mean error distance of approximately 1.32 cm was found when using only nine channels attached on the midline of the motor cortex. It was documented that reliable FDI MEP was observed with an area of approximately 12.9 cm^2^ (3.6 cm $\times$ 3.6 cm), meaning that a reliable MEP could be evoked with a distance of up to approximately 1.8 cm based on the center of the motor hotspot area [37]. Therefore, although the mean error distance of our proposed motor-hotspot-identification method statistically increased as the number of channels decreased, it is expected that our EEG-based machine-learning approach could be utilized by employing only the motor cortex channels for motor hotspot identification, thereby improving its practicality.

The performance of our proposed motor-hotspot-identification method was better when using higher frequency EEG features (delta: 1.20 ± 0.09 cm; theta: 1.17 ± 0.10 cm, alpha: 1.05 ± 0.09 cm, beta: 0.33 ± 0.03 cm, gamma: 0.22 ± 0.03 cm, full: 0.30 ± 0.03 cm). In particular, beta and gamma PSDs showed significantly lower mean error distances than those of delta, theta, and alpha PSDs. Much evidence has been accumulated indicating that a hand motor task significantly changes EEG frequency information in relatively higher frequency bands, i.e., alpha, beta, and gamma bands [28–31, 38–41], and thus the higher performance obtained using higher frequency EEG features can be explained from a neurophysiological point of view. In particular, it has been relatively less documented that gamma frequency band is closely related to motor tasks as compared to alpha and beta frequency bands. Significant changes in gamma power during motor tasks have been mainly reported in ECoG studies [42], but which has been also reported in many EEG studies [29–31]. For example, one study showed a common gamma activity patterns both in ECoG and EEG measurement over the sensorimotor cortex [28]. On the other hand, alpha PSDs did not show better performance as compared to delta and theta PSDs even though alpha band is also closely associated with motor tasks, which should be further investigated in future studies to optimize EEG frequency bands for more accurate motor hotspot identification based on the proposed EEG-based machine-learning approach. Furthermore, because we tested our EEG-based motor-hotspot-identification approach with healthy subjects in this study, further investigation should follow to see whether our results are transferred to patients with neurological disorders in terms of the frequency band used for motor hotspot identification. It has been well documented that EEG spectral powers in relatively higher frequency bands are most closely related to motor functions for healthy individuals as well as patients with neurological disorders. However, note that weaker PSDs are observed over the motor cortex for patients with neurological disorders compared to healthy individuals [43–45]. Therefore, we expect that relatively higher frequency bands would show a better performance of EEG-based motor hotspot identification for patients with neurological disorders similar to the results of this study performed with healthy subjects.

In this study, we used an ANN model to demonstrate the proof-of-concept of the proposed EEG-based motor-hotspot-identification method. Linear and non-linear regression methods were first employed, but they did not show meaningful results (e.g., > a mean error distance of 4 cm). The introduction of other machine learning algorithms would contribute to the performance enhancement of our ANN-based motor-hotspot-identification approach, which could be usefully utilized, especially, when using a reduced number of EEG channels showing relatively lower performance (Fig. 6). In particular, deep learning algorithms could be good candidates to improve the performance of our ANN-based motor-hotspot-identification approach because recent EEG studies demonstrated the possibility of using deep learning algorithms on the performance improvement of various EEG-based applications, such as brain-computer interface, drowsiness detection, mental state monitoring, and so on [46]. We will consider to introduce more advanced machine learning approaches to improve the current performance of ANN-based motor-hotspot-identification approach in future studies.

The difference between the mean error distances for the left and right hands were not statistically significant for most comparison cases except two (i.e., full band and Ch_Set3). This might indicate that our proposed approach is not sensitive to handedness for motor hotspot identification. However, as all subjects recruited in this study were right-handed, additional experiments are required with left-handed subjects to carefully address the mentioned hypothesis.

The application of tES is not only limited to motor rehabilitation but can also be applied to various psychiatric disorders, such as depression, schizophrenia, and attention deficit hyperactivity disorder, to improve not only their cognitive functions, but also neurologically relieve their symptoms [47–51]. In order to apply tES to psychiatric disorders, a target location should be first determined, similarly to motor hotspots for motor rehabilitation. For cognitive rehabilitation, in general, the anodal electrode is attached to F3 according to the international 10–20 system to stimulate the dorsolateral prefrontal cortex (DLPFC), which is known to be associated with various cognitive functions, and the cathodal electrode is attached to F4 or the supraorbital area of the contralateral hemisphere [51–53]. However, as the location of motor hotspots varies from an individual to another, it could be assumed that the tES target location for cognitive rehabilitation is also slightly different between individuals. Therefore, our proposed motor-hotspot-identification approach might be also used for precisely finding a tES target location to maximize the positive effect of cognitive rehabilitation.

Our proposed EEG-based motor-hotspot-identification method used the motor hotspot coordinates identified by TMS to construct an ANN model. If we use our motor-hotspot-identification algorithm in real-world clinics, a patient should register the motor hotspot location identified by TMS for the first time, but after which only EEG is required to identify motor hotspot location for a same subject. This is very useful for patients with impaired mobility (e.g., stroke patients) when periodically using tES for motor rehabilitation, enabling for home-based treatment after a single hospital visit to find an individual motor hotspot location using TMS-induced MEP. Currently, patients visit a hospital for the tES-based motor rehabilitation whenever tES application is required in order to find an individual motor hotspot using TMS before tES application. The motor hotspot information obtained using TMS should be ultimately excluded in the process of finding a motor hotspot using a machine learning technique to further improve the practicality of tES application, and thus we will continuously advance our EEG-based algorithm in such a way that the motor-hotspot-identification algorithm ultimately does not require the motor hotspot information obtained using TMS for a new subject. Despite the mentioned limitation, it is thought that the results shown in this study could prove the proof-of-concept of the proposed EEG-based motor-hotspot-identification method, and they made a meaningful step toward practical tES application.

## Conclusion

In this study, we proposed a novel EEG-based motor-hotspot-identification approach as an alternative to TMS and demonstrated its feasibility via EEG experiments. We also confirmed the possibility of using our proposed method to the development of a practical EEG-based motor-hotspot-identification system with a small number of channels attached only on the motor cortex. Because the brain activity patterns of patients with motor impairment would be different from those of healthy subjects, our proposed motor-hotspot-identification method should be further verified with patients to carefully demonstrate its clinical feasibility.

## Data Availability

Data supporting the findings of this study are available from the corresponding author on request.

## Abbreviations

tES: Transcranial electrical stimulation
NIBS: Non-invasive brain stimulation
tDCS: Transcranial direct current stimulation
tACS: Transcranial alternating current stimulation
tRNS: Transcranial random noise stimulation
TMS: Transcranial magnetic stimulation
DLPFC: Dorsolateral prefrontal cortex
MEP: Motor evoked potential
EEG: Electroencephalography
IRB: Institutional review board
FDI: First dorsal interosseous
EMG: Electromyography
ICA: Independent component analysis
PSD: Power spectral density
FFT: Fast Fourier transform
ANN: Artificial neural network
